# Adult and yearling pampas deer stags (*Ozotoceros bezoarticus*) display mild reproductive seasonal patterns with maximum values in autumn

**DOI:** 10.1590/1984-3143-AR2020-0021

**Published:** 2020-06-29

**Authors:** Rodolfo Ungerfeld, Matías Villagrán, Jorge Gil-Laureiro, Adrián Sestelo, Florencia Beracochea, Fernando Fumagalli, Alejandro Bielli

**Affiliations:** 1 Departamento de Biociencias Veterinarias, Facultad de Veterinaria, Universidad de la República, Montevideo, Uruguay; 2 Laboratorio de Reproducción Animal, Centro Universitario Regional Litoral Norte Salto, Universidad de la República, Paysandú, Uruguay; 3 Laboratorio de Biotecnología Reproductiva, Ecoparque, Buenos Aires, Argentina; 4 Área de Semiología, Facultad de Veterinaria, Universidad de la República, Montevideo, Uruguay

**Keywords:** cervid, electroejaculation, rut, seasonality, sperm

## Abstract

The pampas deer is an endangered species, from which reproductive biology little is known. We aimed to describe and compare the reproductive seasonal patterns of adult and yearling pampas deer stags throughout the year, including morphological traits, testosterone concentration, sperm morphology and cryoresistance pattern changes. Six adult (AS) and five yearling (YS) stags were captured with anesthetic darts once in winter, spring, summer and autumn to study morphological variables, serum testosterone and semen. Adult males were heavier, their neck girth tended to be greater and their testosterone concentration was higher than in YS. Animals were heavier in summer and autumn. Neck girth and testosterone concentration were greater in autumn. Scrotal circumference, testicular volume and gonado-somatic index varied with seasons, decreasing from winter to spring, increasing in summer and remaining in greater values in autumn. Sperm quality had maximum values from summer to winter. However, the cryoresistance ratio of motility score was greater in spring. In conclusion, in the captivity conditions, pampas deer stags seems to present a light seasonal reproductive pattern, with maximum testis size, testosterone secretion and fresh semen quality in autumn. Nevertheless, sperm cryoresistance ratio seemed to remain stable along the year. Although YS were still growing, they achieved similar semen quality than AS.

## Introduction

Reproductive strategies vary widely among deer species as different inter and intra specific patterns were developed to adapt more effectively to their environment. Most cervid species have a seasonal reproductive pattern (see reviews: Drion et al., 2003; Asher, 2011), mainly as a response to photoperiod variation and absolute day length (see review: Zerbe et al., 2012). However, the same species may develop different seasonal reproductive patterns according to the specific environmental conditions, as happens with *Odocoileus* species (Bronson, 1990). Generally, stags start increasing their body weight, neck girth, testicular size, and testosterone concentration 2-3 months before the onset of females’ cyclic activity (Lincoln et al., 1972; Haigh et al., 1984). Associated with those morphological and physiological changes, seminal quality also varies along the year in several deer species (fallow deer: Asher et al., 1996; roe deer: Goeritz et al., 2003; spotted deer: Umapathy et al., 2007; tufted deer: Panyaboriban et al., 2016). Seasonal deer species also present an annual antler cycle closely associated with testosterone plasma concentration: antler completion of growth and mineralization occurs while testosterone concentration increases, and antlers cast 2-4 weeks after testosterone concentration declines (Bartoš et al., 2009).

The pampas deer (*Ozotoceros bezoarticus*) is an endangered South American species, with isolated populations inhabiting Brazil, Argentina, Bolivia and Uruguay. Although the distribution of births along the year suggests that it is a seasonal breeder (Ungerfeld et al., 2008a), there is a paucity of studies referring to its seasonal reproductive changes. The period during which births occur varies according to the location of the population. Births take place mainly in spring in temperate-subtropical populations (Jackson et al., 1980). However, although in a semicaptive population allocated at the Estación de Cría de Fauna Autóctona Cerro Pan de Azúcar (ECFA; Uruguay, 34°S) most births are mainly observed in spring, births can be observed all year round (Ungerfeld et al., 2008b). Nevertheless, in the tropical Pantanal (Brazil, 18° S) −where most environmental changes are related to the alternation of rainy-dry seasons− births are only observed in winter-spring, when vegetation is lusher (Tomás, 1989). In this population, stags exhibit an annual cycle in faecal testosterone concentration, with three peaks reported in summer, early autumn, and winter-spring (Pereira et al., 2005). The rut at the ECFA begins in February and ends in late May (late summer to autumn, (Morales-Piñeyrúa and Ungerfeld, 2012), and stags display a synchronized antler cycle, with antlers casting along a 15-days period (August, mid-winter; (Ungerfeld et al., 2008c). In this allocation, stags have a clear seasonal behavioural pattern: the frequency of agonistic and marking behaviour increases before the onset of the rut, and is greater in adult than in young stags (Delbene and Ungerfeld, 2019).

Interestingly, the seasonal reproductive patterns of pampas deer females allocated at the ECFA differ according to their age. Annual dispersion of births from multiparous females decreases from the second to at least the fourth birth (Ungerfeld et al., 2008b). In this population, first antler stags cast their antlers earlier than adult stags (Ungerfeld et al., 2008c). As in this population males receive similar amounts of the same food all along the year, it seems that the reproductive response of pampas deer stags to other environmental cues might determine different seasonal changes according to age. Consequently, the aim of this study was to describe and compare the reproductive seasonal patterns of adult and yearling pampas deer stags that received similar amounts of food supply throughout the year, including morphological traits, testosterone concentration, sperm morphology and sperm cryoresistance.

## Methods

### Animals and management

The study was performed at the ECFA (34°3’S, 55°1’W; approximately 6 km from the coastline) with two groups of pampas deer stags: six adult (group AS; 4 to 6 y-old) and five yearling stags (group YS; 1.5 y-old). For this study, the two groups were considered as two treatments, and referred as categories throughout the article. Each group was housed in a separate paddock (0.5 ha) since more than one year before the study started. In each paddock animals had free access to abundant native pastures, trees and shrubs, and water. Animals also received approximately 600 g of dairy cow concentrate/deer from Monday to Saturday. All animals were identified by ear tags. Two AS animals died during the second half of the study (summer and autumn, respectively), due to causes unrelated to this study.

All individuals were captured once during the early period of each season: winter (Win; June 23^rd^ to July 3^rd^); spring (Spr; September 30^th^ to October 9^th^); summer (Sum; January 7^th^ to 13^rd^); and autumn (Aut; March 24^th^ to 26^th^) with anesthetic darts fired from a blowpipe (Telinject, California, USA). The anaesthetic management was performed according to Fumagalli et al. (2012).

### Morphological traits

Animals were weighed, and their neck girth, scrotal circumference, as well as the length, width and depth of each testicle were measured. Testicular volume was the sum of the volume of both testicles, which was calculated assuming an ellipsoid shape [(4/3) π.(lenght/2).(width/2).(depth/2)] (Pérez et al., 2013; Ungerfeld et al., 2017). Also, the gonado-somatic index (GSI: testicular volume/body weight, cm^3^/kg) was calculated.

### Blood collection and testosterone measurement

Blood samples were collected with an intravenous catheter placed into the cephalic vein. Samples were centrifuged (1080 g, 20 min), and the serum was stored at -20 °C.

Serum testosterone concentration was determined in the Laboratorio de Técnicas Nucleares (Facultad de Veterinaria, Montevideo, Uruguay) with a solid phase ^125^I radioimmunoassay (Count-A-Count TKTT, Siemens, Los Angeles, CA, USA). The sensitivity was 0.34 nmol/L, and the intra-assay coefficients of variation were 5.7% and 9.2% for low and high controls.

### Semen collection and evaluation

Semen was collected by electroejaculation using a rectal probe (300 mm length x 19 mm width), with three longitudinal electrodes (30 mm in length) (Model 303, P-T Electronics, Oregon, USA) as described by Fumagalli et al. (2012). Semen volume, motility score (scale 0-5), and individual sperm motility were evaluated acording to Beracochea et al. (2014). Sperm concentration was determined and the total number of sperm/ejaculate was calculated. Sperm morphology and acrosome integrity were evaluated after 1:9 semen dilution in 1% glutaraldehyde solution in 0.165 M cacodylate buffer (pH= 7.3), using an optical microscope with phase contrast (x 400). Eosin-nigrosin staining was performed to evaluate alive sperm (Fernández et al., 2013). The total number of ejaculated motile sperm, ejaculated sperm with progressive motility, ejaculated sperm with functional cell membrane, and ejaculated morphologically normal sperm were calculated.

### Sperm cryopreservation

Semen samples were diluted with a commercial extender (Red extender, IMV Technologies, L'Aigle, France) with 4% of glycerol and 10% of egg yolk. Then, semen samples were plugged in straws and placed in a container with water (approximately 1000 mL at room temperature), which was introduced into a freezer (-10 °C) during 90 min until reaching 5 °C. The straws were then maintained at 5 °C for 2 h, and the samples were placed in nitrogen vapors (5 cm above the surface of a cryostorage box) for 10 min before plugging them into liquid nitrogen. Frozen samples were thawed at room temperature for 10 s, immediately placed in a water bath at 37 °C for 30 s, the content was poured into an Eppendorf tube and maintained at 37 °C. Motility score, percentages of motile sperm and sperm with progressive motility, with normal morphology and with integral acrosome were determined after thawing.

The response to cryopreservation for the motility score, percentage of motile sperm, sperm with progressive motility and morphologically normal sperm was calculated as a cryoresistance ratio (CR) (Esteso et al., 2006), as the Value after thawing X 100 / Value before thawing.

### Statistical analysis

All data were compared with a mixed model including stags’ category (AS vs YS), season (winter, spring, summer and autumn), as well as the interaction between category and season as main effects, and the individual within each category as a random effect. Data are expressed as LSMeans ± SEM.

### Registration and ethics committee approval

The study was approved by the Comisión Honoraria de Experimentación Animal, Facultad de Veterinaria (Universidad de la República), and in accordance with the ARRIVE guidelines and the U.K. Animals (Scientific Procedures) Act, 1986 and associated guidelines, EU Directive 2010/63/EU (European Union, 2010) for animal experiments, or the National Institutes of Health guide for the care and use of Laboratory animals (NIH Publications No. 8023, revised 1978).

## Results

### Morphometric traits and testosterone concentration

**Figure 1:**
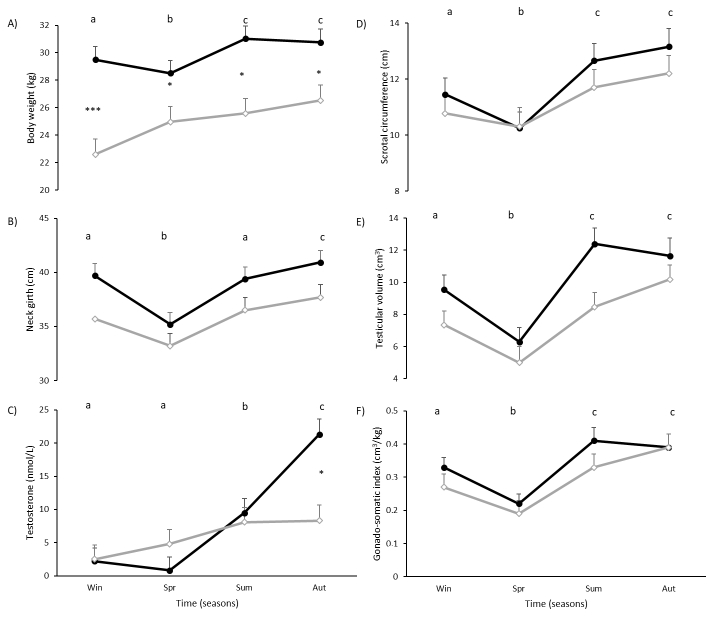
Body weight (A), neck girth (B), testosterone concentration (C), scrotal circumference (D), testicular volume (E) and gonado-somatic index (testicular volume/body weight) (F) in adult (–⬤–) and yearling (–◇–) pampas deer (*Ozotoceros bezoarticus*) males during different seasons in Uruguay (SH). Different letters: P<0.05 for differences in time. *P<0.05; ***P<.0001: Differences between adult and yearling males at the same time.

Effects of main factors included in the model (category, season, and their interaction) are presented in Table 1. Adult stags were heavier than YS stags (30.0 ± 0.9 kg vs 24.9 ± 1.1 kg, P=0.005, respectively). Body weight varied with time (P<0.0001), increasing from Win to Spr (P=0.048), and from Spr to Sum (P<0.0001) (Figure 1A). There was also a significant interaction between category and time (P=0.0004): AS were heavier than YS in all four seasons (Win: P=0.0002, Spr: P=0.025, Sum: P=0.001 and Aut: P=0.01). Neck girth tended to be greater in AS than in YS (38.8 ± 0.9 cm vs 35.8 ± 1.1 cm respectively; P=0.06). It varied with time (P<0.0001): it decreased from Win to Spr (P<0.0001), increased in Sum (P<0.0001), and again in Aut (P=0.01) (Figure 1B). There was no interaction between category and time.

**Table 1:** Main effects of age (adult vs yearling stags), time (seasons) and their interaction in morphometric values and testosterone concentrations in pampas deer males.

	**Age**	**Time**	**Age** ^*^ **Time**
Morphometric data			
Body weight	0.005	<0.0001	0.0004
Neck perimeter	0.06	<0.0001	ns
Scrotal circumference	ns	<0.0001	ns
Testicular volume	0.037	<0.0001	ns
Gonado-somatic index	ns	<0.0001	ns
Testosterone	0.025	<0.0001	0.013

ns: non-significant.

* Interaction age and time.

Testosterone concentration was greater in AS than in YS (8.5 ± 0.7 nmol/L vs 5.9 ± 0.7 nmol/L respectively; P=0.025). It varied with time (P<0.0001): concentration increased from Spr to Sum (P=0.017), and from Sum to Aut (P=0.024) (Figure 1C). There was also a significant interaction between category and time (P=0.013): testosterone concentration was greater in AS than in YS in Aut (P=0.0007).

Scrotal circumference varied only with time (P<0.0001): it decreased from Win to Spr (P=0.04), increased in Sum (P<0.0001), and remained in greater values in Aut (Figure 1D). Testicular volume in AS was bigger than in YS (9.96 ± 0.64 cm^3^ vs 7.75 ± 0.64 cm^3^ respectively; P=0.037), and varied with time (P<0.0001) (Figure 1E). Similarly, to scrotal circumference, it decreased from Win to Spr (P=0.002), increased in Sum (P<0.0001), and remained in greater values in Aut. The GSI only varied with time (P<0.0001), with the same general pattern as scrotal circumference and testes volume: it decreased from Win to Spr (P=0.004), increased in Sum (P<0.0001), and remained in greater values in Aut (Figure 1F).

### Semen variables

#### Fresh semen

Semen was collected from all the animals. Effects of main factors included in the model (category, season, and their interaction) are presented in Table 2. There were no effects of males’ category or interaction of category and season for any variable. There were seasonal variations in semen volume, sperm concentration, motility score, the percentages of sperm with progressive motility, alive sperm, and morphologically normal sperm (P=0.021; P=0.026; P=0.005; P=0.011; P=0.032; P=0.001 respectively).

**Table 2:** Main effects of age (adult vs yearling stags), time (seasons) and their interaction in seminal parameters in pampas deer males.

	**Age**	**Time**	**Age** ^*^ **Time**
Volume	ns	0.021	ns
Concentration	ns	0.026	ns
Mass motility	ns	0.005	ns
Percentage of:			
Motile sperm	ns	ns	0.078
Sperm with progressive motility	ns	0.011	ns
Alive sperm	ns	0.032	ns
Morphologically normal sperm	ns	0.001	ns
Total number of:	ns	ns	ns
Ejaculated sperm	ns	0.068	ns
Motile sperm	ns	0.060	ns
Sperm with progressive motility	ns	0.073	ns
Alive sperm	ns	ns	ns
Morphologically normal sperm	ns	0.10	ns

ns: non-significant.

* Interaction age and time.

**Figure 2:**
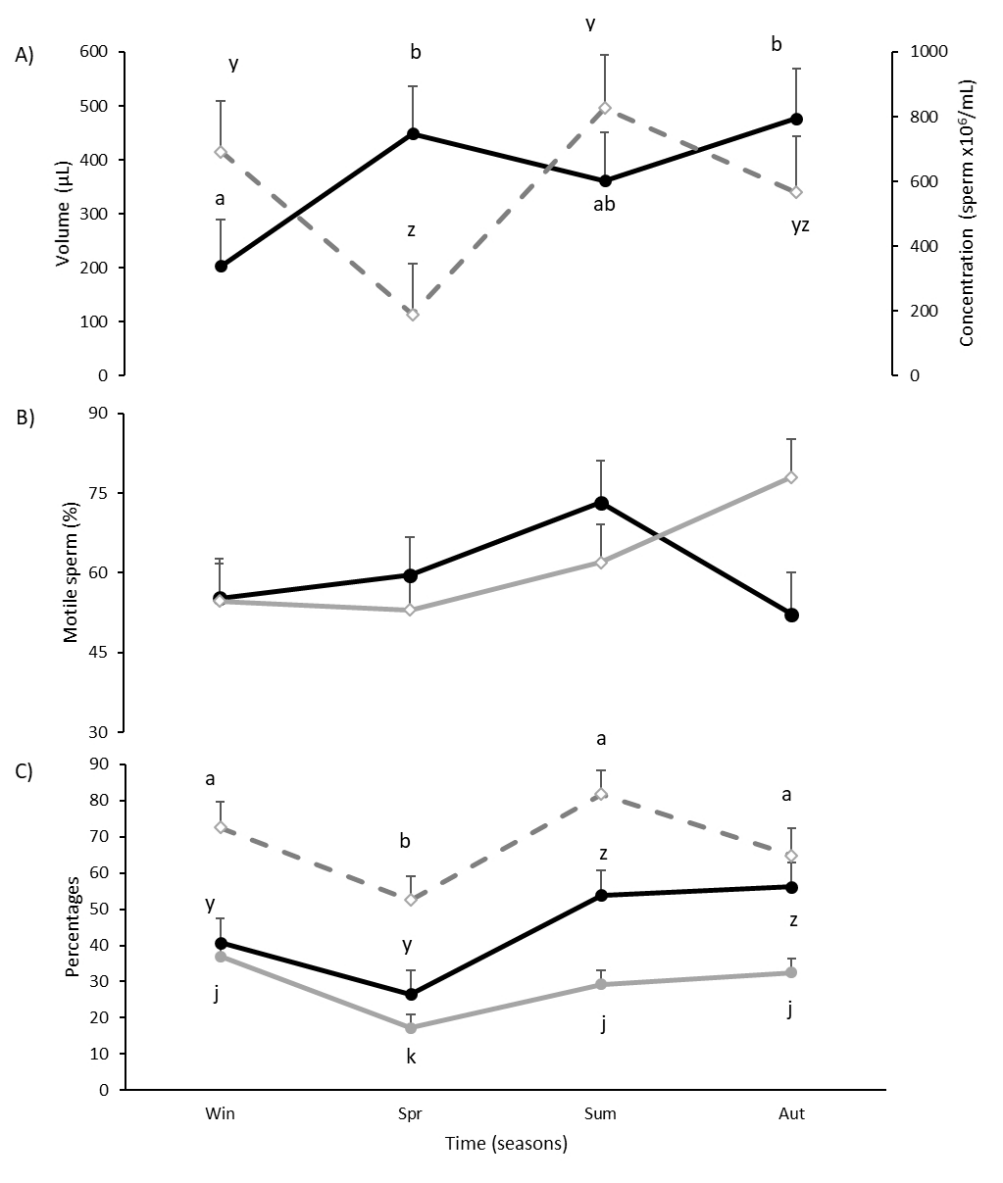
Semen characteristics in pampas deer (*Ozotoceros bezoarticus*) males during different seasons in Uruguay (SH): (A) semen volume (–⬤–) and concentration (--◇--); (B) percentage of motile sperm in adult (–⬤–) and yearling (–◇–) males; (C) percentages of alive sperm (--◇--), motile sperm (–⬤–), and morphological normal sperm (–⬤–). Different letters indicate significant differences in time (P<0.05) for each line of the graphic.

Semen volume increased from Win to Spr (P=0.008), tended to decrease in Sum (P=0.085), and remained at higher values in Aut (Figure 2A). Semen concentration decreased from Win to Spr (P=0.019), increased again in Sum (P=0.005) and tended to decrease in Aut (P=0.084) (Figure2A). There was only a tendency for an interaction in the percentage of motile sperm (P=0.078) (Figure 2B). The percentage of sperm with progressive motility increased from Spr to Sum (P=0.006), and remained in similar values in Aut (Figure 2C). The percentage of ejaculated alive sperm decreased from Win to Spr (P=0.047), increased again in Sum (P=0.005), and tended to decrease in Aut (P=0.1) (Figure 2C). The percentage of morphologically normal sperm decreased from Win to Spr (P=0.0003), increased in Sum (P=0.010), and remained high in Aut (Figure 2C).

The total number of ejaculated sperm (174.9 X 10^6^ ± 37.1, P=0.068), ejaculated motile sperm (125.5 X 10^6^ ± 28.0, P=0.06), ejaculated sperm with progressive motility (107.1 X 10^6^ ± 25.7, P=0.073) and ejaculated morphologically normal sperm (67.5 X 10^6^ ± 17.3, P=0.1) tended to vary with seasons. The total number of ejaculated alive sperm was 154.0 X 10^6^ ± 33.9 sperm.

#### Thawed semen

**Figure 3:**
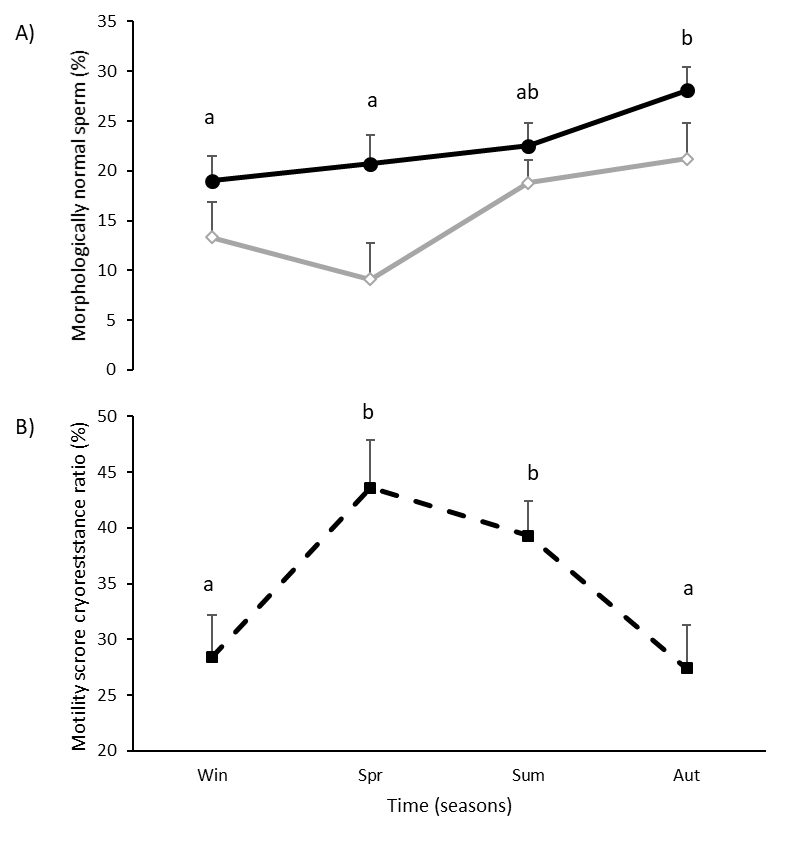
(A) Percentage of thawed sperm with morphological normal sperm in adult (–⬤–) and yearling (–◇–) males; (B) cryoresistance rate of sperm motility score in pampas deer (*Ozotoceros bezoarticus*) males. In **Figure 3B** data from adult and yearling males presented pooled as no differences were observed. Different letters indicate significant differences in time (P<0.05) for each line of the graphic.

Only the percentage of morphologically normal sperm varied according to males’ age: it was greater in AS than YS (22.6 ± 1.8% vs 15.6 ± 1.4%, P=0.014). It also varied between seasons (P=0.029), increasing from Win to Aut (P=0.01) (Figure 3A). The percentage of motile sperm tended to vary with time (P=0.073), and it also tended to vary as consequence of an interaction between stag category and seasons (P=0.075). The only cryoresistance ratio with significant effects was the motility score, which varied with time (P=0.031): it increased from Win to Spr (P=0.022), remained in greater values in Sum, and decreased in Aut (P=0.034) (Figure 3B).

## Discussion

Pampas deer stags show a clear seasonal reproductive pattern, with strong changes in all morphological variables and testosterone concentration, and less intense changes in seminal quality. On the other hand, slighter changes were observed in relation to stags’ age, as only body weight, testicular volume and testosterone concentration varied along the seasons. The observed seasonal reproductive pattern was probably mainly determined by photoperiod, as food supply was offered homogeneously throughout the year. Other body traits as neck girth also varied in relation to testosterone concentration, as it was previously reported in other deer species (Lincoln et al., 1972). In general, under these conditions, the maximum reproductive activity occurred in summer-autumn, when daylight hours were decreasing, and the lowest activity occurred in spring. However, most values suggest that although stags showed a clearly seasonal pattern, they were able to breed at any time of the year. This result is in agreement with the seasonal pattern of births reported in females from the same population (Ungerfeld et al., 2008b). Although animals from this population are bred in very particular conditions, with several generations also bred in semicaptivity, in some free-living populations at similar latitudes, births can also occur throughout the year (Uruguay: Jackson et al., 1980); Argentina: (Jackson and Langguth, 1987). Overall, at least when food availability is not restricted, it seems that pampas deer seasonal pattern can be flexible, and thus, extended to other periods of the year. The recrudescence in stags’ reproductive pattern - including testis size, testosterone concentration and semen quality - occurred 2 to 3 months before the rut (Morales-Piñeyrúa and Ungerfeld, 2012), as it was expected in a seasonally breeding ruminants (Lincoln and Short, 1980).

The lack of a strong seasonal pattern, and especially the slight differences observed in sperm quality according to males’ category and to the moment of the year open interesting possibilities for the application of reproductive biotechnologies. In general, sperm collection and cryopreservation is a bottleneck for most reproductive biotechnologies, so the wide period and age in which sperm can be collected with similar results considered together with the low risks of handling the animals (Fumagalli et al., 2012) allows to collect semen in a wide period. Other environmental factors such as the contact with females (Villagrán and Ungerfeld, 2013) or the hierarchical individual position of each male (Villagrán et al., 2018) seem to have greater impact than seasonality or males’ category on some characteristics of the ejaculate, which should be considered for the instrumentation of reproductive biotechnologies.

Body weight of these stags also varied seasonally despite they had free access to similar amounts of ration throughout the year. Therefore, body weight variations could be associated with either seasonal changes in thermoregulatory energetic demands (Fourier et al., 1999), the cost of antler growth (French et al., 1956) and/or seasonal fluctuations in deer appetite (with lowest appetite in winter (Putman, 1988)). Alternatively, the decrease in body weight in AS stags in autumn could be consequence of the extra energy expenditure provoked by the peak in agonistic and marking activity (Delbene and Ungerfeld, 2019). On the other hand, YS continued growing throughout the studied period: it should be considered that although these stags had attained their puberty before the study began, they had not finished growing when our study ended (Ungerfeld et al., 2011). Interestingly, gonadal changes were independent from body weight changes, as the GSI seasonal pattern was similar to the testicular volume pattern. Moreover, although YS continued growing during winter, their testis size decreased similarly as in AS. This agrees with previous reports in other small ruminants i.e., rams, whose testes also reduce their size in winter even while their body weight is increasing (Ungerfeld, 2012). However, the difference in testes size between AS and YS is in agreement –and thus, probably related- with differences in their body weight. The slow growth rate of pampas deer stags, that had not ended when 2.5-y-old, is in agreement with reports for the development of rams in the same region (Ungerfeld, 2012; Pérez et al., 1997). This confirms that the general reproductive pattern in moderately seasonal small ruminants are similar during the developmental period.

It is interesting that testosterone seasonal patterns differed between AS and YS. While in AS it followed the same seasonal pattern as other traits, in YS there was not a winter decrement in testosterone concentration. However, it should be considered that due to difficulties in animal manipulation, only one sample/animal was collected in each season, which hampers attaining definitive conclusions regarding this pattern. All stags reduced their neck girth during winter and spring. The neck girth is a secondary sexual trait of stags, stimulated by the anabolic action of testosterone (Handelsman, 2006). Other authors provide a non-opposed explanation, as they relate this increase to muscular exercise performed during fights between stags before the rut (Clutton-Brock et al., 1982).

Semen variables changed seasonally similarly to morphological traits. However, it is interesting that effects were stronger in semen quality – as most variables were affected – than in the total sperm ejaculated. As semen quality is an important limitation for semen cryopreservation, it can be recommended not to collect semen for cryopreservation in winter. On the other hand, although there were seasonal changes, semen quality was similar from summer to winter, backing the concept of weak seasonal effects with a relatively long breeding period. Moreover, the values in the ejaculate only tended to vary along the year, and there were only slight seasonal changes in thawed sperm, supporting the concept that the pampas deer is a species with light seasonality. This may be important given the general low semen quality reported in pampas deer (Beracochea et al., 2014). Alternatively, the long period during which pampas deer stags (this study) and females (Ungerfeld et al., 2008d) can reproduce may be useful at least in subtropical regions to minimize the possible effects of low semen quality on fertility in ex-situ breeding.

The semen seasonal pattern seems to be associated to the other traits determined in this study. Although the testosterone pattern is less clear, this may be consequence of collecting only one sample per season in a low number of animals. Testosterone is closely related to sperm production and quality in other deer species (roe deer: (Blottner et al., 1996); wapiti: (Haigh et al., 1984)). A direct relationship between testosterone concentration and germ cells production in the seminiferous tubules, sperm numbers in the cauda epididymidis, and semen quality has been reported previously (Loudon and Curlewis, 1988; Monfort et al., 1993)). However, although AS reached greater testosterone concentration in autumn, this did not triggered differences in semen quality. Moreover, motile sperm tended to vary differently in AS and YS according to seasons, including greater values in YS in autumn. It should be considered that differences in testosterone concentration may have greater impact 2 to 3 months later, according to the period of spermatogenesis and epididymal maturation in most ruminants.

Yearling stags were still growing, and according to previous data, did not reach their maximum antler size (Ungerfeld et al., 2008d) and testosterone concentration (Ungerfeld et al., 2011) until they were 6 years old. However, semen characteristics were similar to those of adults as early as 1.5 years old. Considering that pampas deer females have a very short period of receptiveness in each oestrous cycle (only some minutes: Morales-Piñeyrúa and Ungerfeld, 2012), and probably YS have less opportunities to access to females, the precocious semen maturation may be essential to increase their probability of letting offspring through opportunistic matings.

## Conclusion

In conclusion, in the captivity conditions, pampas deer stags seems to present a light seasonal reproductive pattern, with maximum testis size, testosterone secretion and fresh semen quality in autumn. Nevertheless, sperm cryoresistance ratio seemed to remain stable along the year. Although YS were still growing, they achieved similar semen quality than AS.
